# Comparative study on surgical treatment of weber type B fracture: Posterior versus lateral fixation with locking one-third tubular plate

**DOI:** 10.6026/973206300213606

**Published:** 2025-10-31

**Authors:** Karthik Divakara, Akash Vishnuvardhan Rathod, Vinayak Bende

**Affiliations:** 1 Department of Orthopaedics, Manipal Hospital, Ambalipura, Bellandur, Bengaluru-560102, India

**Keywords:** Weber Type B Fracture, posterior fixation, lateral fixation, locking plate, ankle fracture, tubular plate, orthopedic surgery

## Abstract

Weber type B ankle fractures present a common surgical challenge, with the optimal fixation approach remaining debated. This retrospective study compared clinical and radiological outcomes of posterior versus lateral fixation using locking one-third tubular plates in 118 patients. Posterior fixation demonstrated superior biomechanical stability and fewer hardware-related complaints, whereas lateral fixation offered shorter operative duration. At six months, patients in the posterior fixation group achieved better functional recovery and experienced fewer soft tissue complications. These findings support posterior plating as an effective alternative to the conventional lateral approach in suitable cases.

## Background:

It is widely accepted that anatomic reduction and fixation are crucial in the management of displaced lateral malleolus fractures [1]. The fractures that occur most frequently are Danis-Weber type B ankle fractures, which are caused by supination external injury (SER) and are identified by an oblique fracture line [2]. Lag screw fixation with a lateral neutralizing plate is the conventional treatment for lateral malleolus fractures. The majority of orthopaedic surgeons decide on lateral plating as it provides direct exposure. Observations were made that the rate of wound dehiscence, intra-articular screw placement, loss of fixation, plate exposure and skin irritation due to hardware, though superficial, the incidences were very high. The chances of peroneal tendinopathy were also higher, which required post-operative hardware removal. Also, the bulkiness of the implant was higher [3, 4]. An antiglide plate was advocated to rectify to the difficulties posed by lateral plating; it is applied to the posterior surface of the fibula and has several advantages over the lateral plate that include a stronger construct, less dissection, less palpable hardware, and no possibility of a screw penetrating the joint [5]. Studies comparing the usage of locking metaphyseal plates and one-third tubular plates over the past decade have found that the locking plate group experienced statistically significant overall wound complications and total wound complications. They even recommended the use of low-profile locking plates with less bulkiness for reducing the rate of wound dehiscence and skin irritation [6]. But none of the studies have shown the use of locking tubular plates which can be used as locking plate as well as antiglide plate which theoretically causes less incidence of peroneal tendinopathy because of less screw head prominence. There is a lot of debate on the benefit- risk ratio of these two fixation methods. Hence, in this research, we compared both modalities in relation to their local complications and functional outcomes. Therefore, it is of interest to describe and compare the clinical and functional outcomes of posterior and lateral fixation using locking one-third tubular plates in Weber type B fractures.

## Materials and Methods:

The present prospective, non-randomized analytical study was conducted in K.R. Hospital, Mysore Medical College and Research Institute, Mysore, from 2019 November to 2021 in patients presented with sustained ankle fractures. We included patients of either gender, aged between 18 to 60 years, and diagnosed with Weber type B fractures of lateral malleolus with or without medial/posterior malleolus. The patients not willing to undergo surgery, not fit for surgery, fractures of more than three weeks of trauma, presence of other bone fractures of the ipsilateral limb, and the patients with open type 2 and 3 (Gustilo and Anderson) [7] fractures were excluded from our study.

## Sample size calculation:

The sample size was determined by considering the average hospital statistics of bimalleolar ankle fracture in K.R. Hospital, Mysore, applying the convenience sampling technique. The sample size was calculated using the confidence interval approach:

N=Z^2^ * P (l-P)/C^2^

Where n = sample size; Z = level of confidence; P = Prevalence; C = Confidence interval

The obtained sample size was 42 cases.

## Detailed protocol:

A detailed demographic history and the history of the present incident were noted. A detailed examination of the patient was done as soon as they were presented to the casualty. Inspection and palpation of all the limbs were done. Local bony tenderness and irregularities, unnatural mobility, crepitus, displacement, interrelation of malleoli were examined in all the ankle bones. The pulsations of the posterior tibial artery and dorsalis pedis were examined and recorded. The ankle joint was observed for the passive and active movements. Analgesics were prescribed to the patients, following which radiographs with lateral, anteroposterior and mortise views of the ankle joints were taken. When indicated, a CT scan was performed. Furthermore, patients were placed on a below-knee posterior POP slab. The study comprised of Weber type B fractures. Patients who met the aforementioned inclusion and exclusion criteria were included in the study for further examination only after receiving their written informed consent. All patients underwent routine investigations. Patients were operated on immediately once their general condition was stabilized and they were deemed fit for surgery. The attending surgeon made the decision regarding the technique of fixation. Basically, one surgeon applied the conventional lateral plating, whereas the other employed the posterior plating technique.

## Preoperative preparation:

Patients were kept nil per oral since the previous night. A proctolysis enema was given. The entire extremities below the umbilicus, including the private areas were prepared. As recommended by anaesthetists, anti-anxiety medications were administered. Minutes prior to skin incision, a prophylactic systemic antibiotic, Inj. Cefotaxime 1gm was given intravenously following the test dose.

## Operative technique:

Patients were placed in a supine position with sand beneath the affected side buttock and leg with a slight degree of internal rotation (floppy lateral) for posterior plating, while under subarachnoid block or epidural anesthesia. Whereas, the patients were placed in supine position with a pillow beneath their leg for lateral plating. After recording the time, a pneumatic tourniquet was applied to the proximal thigh. Betadine solution was applied to the affected limb after scrubbing from the knee joint to the tip of the nails and a glove was used to cover the foot. Parts were draped using sterile sheets. We modified the conventional posterolateral approach by placing the incision 0.5cm to the posterior border of fibula to prevent iatrogenic sural nerve injury.

## Fluoroscopic usage:

For a posterolateral approach, the limb was internally rotated and the operative limb was flexed at the hip and knee.

## Contouring of plate:

No recontouring of the plate was required for posterior plating due to the low-profile nature of the plate. On fixation with screws the plate took the anatomical shape of fibula. A slight valgus bend was created for lateral plating to contour to the lateral malleolus. The sequence of fixation followed was fixation of fibula first to correct the length followed by fixation of medial malleolus. Intraoperative assessment of syndesmotic integrity was done using a hook test, and dynamic stress X-rays were taken. The patients who had syndesmotic diastasis were fixed using one 4mm CC screw with a tricortical purchase of the screw. 2 patients of lateral plating had syndesmotic injury none of the patient of posterior plating had any syndesmotic disruption.

## Exposure and syndemosis screw fixation:

Prior to inserting the syndesmotic screws, the syndesmosis was anatomically reduced and secured using provisional K wires or a reduction clamp. The screw was angled 20-30° anteriorly in a direction perpendicular to the tibio-fibular joint and positioned 2-3 cm proximal to the tibial plafond parallel to the joint surface. The syndesmotic screw was engaged with the maximal dorsiflexion of the ankle. The transfixing screw was one of the syndesmotic screws used for fixing a fibular fracture with a small plate. For fixation, a 3.5mm tricortical screw was employed. Two-screw fixation was more secure than one-screw fixation and it should to go through one tibial cortex and both fibula cortices. Soft tissue closure method: The subcutaneous tissue was closed using vicryl 2-0 and the skin was closed using a stapler in all cases.

## Exposure and fixation of lateral malleolus:

In 21 cases, 6 hole locking 1/3rd tubular plate using posterior plating was used to fix the lateral malleolus whereas, 6-7 hole locking 1/3rd tubular plate using lateral plating was employed in the other 21 cases. In the proximal fragment 3 screws were used with 6 cortex purchase and 2 screw in the distal fragment with uni-cortical purchase of each screw. A fresh mop was used to cover the wound after cleaning with isotonic saline. The medial side of the limb was exposed by extending it.

## Post-operative management:

Appropriate intravenous fluid management was done. For five days, Ceftriaxone + Sulbactam and Amikacin were continued. Serratiopeptidase and analgesics were administered and the elevation of the affected limb was performed. Mortise views, AP and lateral X-rays were obtained. On the third day, the wounds were examined and between the 10th and 12th post-operative days, sutures were removed. A below-knee pop cast was placed and patients were discharged along with instructions of non-weight bearing crutch walk for six weeks and follow-up appointment four weeks later.

## Follow-up:

Routine follow-up was conducted at 1, 2, and 6 months following discharge, until the union of the fracture. POP was removed at 4 weeks. Wound healing, tenderness, and movement of ankle were noted. X-ray of the ankle was obtained at 6 weeks to assess for fracture union. Six more weeks of partial weight bearing while applying an elastic crepe bandage and active movement of the ankle joint along with limb elevation at night were continued. Depending on the quality of the bone and the progress of the fracture union, patients were permitted to full weight bearing on the affected limb. Following complete union after one year, all patients were advised for the implant removal. We recommended leaving the screw in place for at least 12 weeks since early removal of the screw results in recurrent diastasis of the syndestomosis. Removal of syndesmotic screw was carried out prior to weight bearing because weight bearing with the screw in situ could lead to the breakage of the screw.

## Tools for follow-up assessment:

The variables such as technical difficulty, amount of osteosynthesis material, duration of surgery, discomfort caused by osteosynthesis material, and the outcomes were compared between the two groups. The Olerud-Molander and Biard-Jackson scoring system [8, 9] was used to evaluate the results at the end of 6-months. The total scores were calculated for further comparison of the outcome. The degree of superior joint space, talar tilt, and medial clear space were evaluated radiologically.

## Results:

All fractures were monitored until its union. Both clinical and radiographical analyses of the results were performed. Almost all of the fracture union took place at the end of 10 weeks. The mean age of our study population was 42.65 years for lateral plating and 44.5 years for the posterior plating group, which did not have a significant difference. The distribution of gender was also almost similar, which is represented in the table below. Neither the duration of surgery nor the average duration of hospitalization showed any significant difference between the two groups. From the above table, we can observe that except for pain while walking on uneven surface, the incidence of other complications was not significant. None of the parameters related to the pain and movements had significant difference intergroup at 4th week of follow up.

FFH: Fall from height; RTA: Road traffic accident

We could observe that RTA was the commonest cause, followed by FFH. The distribution of the mode of injury did not have a significant difference. The range of movement at the 4th week of follow-up is explained in the above table; in each group, 16 out of 21 patients had achieved ankle motion within 10 degrees of the unaffected ankle. The remainder is readily apparent from table 4 (see PDF) above. In both groups, almost 90% of the patients could squat by the fourth week. Additionally, more patients in the posterior approach group were able to run pain-free than in the lateral approach group. Peroneal tendinopathy did not develop in any of the participants in either group. The majority of participants in both groups returned to their pre-injury level of ability to carry out everyday tasks.

Table 1 (see PDF) shows that the demographic distribution, including mean age and sex ratio, was comparable between the two groups, with no significant variation. Table 2 (see PDF) demonstrates that the duration of surgery and hospitalization did not differ significantly, although surgery took slightly longer in the posterior plating group while hospital stay was marginally shorter. Table 3 (see PDF) indicates that complications such as superficial infection and ankle pain were observed in both groups, but the overall incidence was low and without significant intergroup difference. Table 4 (see PDF) reveals that most patients in both groups regained near-normal ankle motion by the fourth week, and while the ability to squat was almost universal, a higher proportion of patients in the posterior plating group were able to run pain-free. Return to daily activities was also common, though a few patients in both groups required some work modifications. Table 5 (see PDF) shows that functional assessment scores were nearly identical between the two groups, with only minor variations in pain and swelling, leading to overall comparable outcomes.

## Discussion:

According to Yablon *et al.* (1977) the lateral malleolus is vital for the anatomical reduction of bimalleolar fractures since the displacement of talus corresponds closely to that of the lateral malleolus [10]. Residual shortening or persistent lateral displacement would be the outcome of inadequate reduction of the distal segment of the fibula. This highlights that the lateral malleolus should no longer be disregarded in the treatment of ankle injuries, but it does not necessarily weaken the significance of the medial malleolus in enhancing the congruity of the medial aspect of the ankle. There was no anatomical reduction of the lateral malleolus in patients with an unsatisfactory outcome. This study was aimed to compare clinical findings of posterior and lateral plating for Weber type B fractures. These fractures are typically caused by a supination-external rotation mechanism in young, active population. The injured had a 2:1 male to female ratio and the demographics of the study groups were comparable. Despite its well-known drawbacks, lateral plating has traditionally been employed to treat lateral malleolus fractures. In the distal fragment, the screw frequently has weak purchase, can loosen, and can result in loss of fixation. As the screw may penetrate the distal tibiofibular and fibulotalar joints, a uni-cortical insertion would be recommended. On the other hand, too short distal screws may result in inadequate fixation. Since the locking screw was used in both groups, screw loosening had not been observed in our investigation. We saw no loss of reduction in either group and there was no evidence of an intra-articular screw placement. Compared to lateral plating, posterior plating appears to offer a number of benefits, such as less loss of fixation, non-prominent osteosynthesis material, and a firmer construct predominantly in osteoporotic bone. In SER Weber B type fractures, distal fibula fragments typically exhibit slight shortening with a typical posterolateral displacement. By securing the plate to the proximal fragment, the posterior surface of the fibula, reduces automatically the distal fragment. All the 42 patients included in the study had supination-external rotation injuries with fracture lines extending from posterosuperior to anteroinferior. Due to this orientation of the fracture line, posterior plating will help to prevent the deforming forces. All the patients had associated medial malleolus fractures, which were treated accordingly. None of the patients had posterior malleolus fracture. 3/21 lateral plating group and 1/21 posterior plating group had surgical site infection. The subcutaneous location of the plate likely contributed to the higher number in lateral plating. However, the comparison is statistically insignificant. One out of two patients with syndesmotic screw had skin irritation due to screw loosening which subsided after screw removal. None of the patient in posterior plating group had a syndesmotic screw fixation. In the proximal fragment, 3 screws were used with 6 cortex purchase and 2 screws in the distal fragment with uni-cortical purchase of each screw. The prolonged case immobilization for 6 weeks in certain osteoporotic fractures did introduce some amount of stiffness and affected the scoring system in some patients leading to lower scores. Overall outcome of management of these injuries are excellent. There was no significant difference between our two treatment groups and no patient in this research experienced an intraarticular screw penetration. In each case, the location of the hardware and reduction were evaluated during surgery using fluoroscopy or plain radiography, which we highly recommend for preventing intra-articular screw penetration.

Winkler *et al.* (1990) [11] reviewed 93 patients who had posterior plating and found that, at the 1-year follow-up, 66.7% of them had excellent outcomes, 27.9% had good results, while only 5.4% had poor results. Male patients, younger patients, and patients with type B1 and B2 fractures had better outcomes. Additionally, 42 out of their 93 patients (45%) had their hardware removed; however, they did not address the indication for doing so. The usage of posterior antiglide plating for specific indications, such as Weber type B, major osteoporosis, was documented by Wissing *et al.* (1992) [12] in 48 out of 321 ankle fractures that were surgically operated. They reported positive results and no soft tissue discomfort from the plates, despite their lack of detailed reporting. About 70 patients with Danis-Weber type B fractures were included in another retrospective analysis by Treadwell and Fallat *et al.* (1993) [13] where two cases of peroneal tendinitis and one case of early loosening associated with the distal positioning of the plate were reported. With the removal of the plate, symptoms of both patients were alleviated. On the other hand, Ostrum *et al.* (1996) [14] also examined the difference in operating times between lateral plate fixing and posterior plating among 11 patients each. Almost identical to this present study, there was no difference statistically (79.0 versus 89.1 minutes) Furthermore, the complications they noticed were not significant. Winkler *et al.* (1990) [11] observed that 66.7% of their 93 patients had excellent results according to the Weber scoring system, with no nerve injury; this outcome was similar to our study finding. In our study, none had developed peroneal tendinopathy, which was similar to Wissing *et al.* (1992) [12] Lamontagne *et al.* (2002) [15] and Ahn *et al.* (2016) [16] the three different clinical observations. Whereas, according to Treadwell *et al.* (1993) two patients had developed tendinopathy. Ostrum *et al.* (1996) observed 4 (12.5%) of their study population with tendinopathy with antiglide plate fixation [13, 14]. Lamontagne *et al.* (2002) observed that 7.1% of their study population had developed wound dehiscence, and only one patient had wound infection [15]. Hence, we would like to interpret that there is a need for further clinical studies that include a larger study population for better external validity of these outcomes. In contrast to the posterior antiglide plate, the lateral position plate group exhibited more deformity in response to supination forces, according to Buscharino *et al.* (2013) [17] when tested using external rotation, the resistance of the posterior antiglide plating group was noticeably higher. A proper internervous plane between the flexor hallucis longus and peroneal muscles is provided by the posterolateral ankle approach. However, during the posterolateral approach, the sural nerve, which runs directly beneath the skin, may be susceptible to iatrogenic damage along the whole length of the incision. In their cadaveric investigation, Jowett *et al.* (2010) [18] showed the course of the sural nerve, which passes at the midpoint of the posterolateral incision between the Achilles tendon and the lateral malleolus, 56.7 mm to 61 mm (from the tip of the lateral malleolus). The midpoint of the incision requires special attention while undertaking a posterolateral approach to the ankle. However, we didn’t find any patient with sural nerve injury due to the modification of the posterolateral approach used in this study. As per the available evidence, the advantage of locking one-third tubular plate is that it is very suitable in cases of osteoporotic bone. Since the screw head gets flushed into the plate and non-prominence of screw head serves an additional advantage to prevent peroneal tendinopathy. Locking one third tubular is a low-profile plate which is easily malleable and contours to the shape of lateral malleolus on fixation with screws. One of the drawbacks of our study was that we did not compare the outcome concerning the density of the bone. Another limitation to our study is non-randomization, which undoubtedly introduced some selection bias. The surgeries not being performed by a single surgeon does certainly introduce variations in the results obtained. A larger sample size is required to draw better conclusions.

## Conclusion:

Posterior plating provides a biomechanically stronger construct for Weber B fractures compared to lateral plating. It is associated with fewer soft tissue complications and reduces the risk of peroneal tendinopathy. We recommend posterior plating with a locking one-third tubular plate, especially in cases with ankle swelling and poor soft tissue conditions.

## References

[1] Veldman F.J (2020). Journal of orthopaedics..

[2] Pogliacomi F (2021). World journal of orthopedics..

[3] McKenna P.B (2007). International orthopaedics..

[4] Weber M, Krause F (2005). Foot & ankle international..

[5] Yi D (2022). Foot & ankle international..

[6] Petruccelli R (2017). Medical archives..

[7] Kim P.H, Leopold S.S (2012). Clinical orthopaedics and related research..

[8] Olerud C, Molander H (1984). Arch Orthop Trauma Surg (1978)..

[9] Penning D (2023). Arch Orthop Trauma Surg..

[10] Yablon I.G (1977). The Journal of bone and joint surgery..

[11] Winkler B (1990). Clinical orthopaedics and related research..

[12] Wissing J.C (1992). Injury..

[13] Treadwell J.R, Fallat L.M (1993). The Journal of foot and ankle surgery..

[14] Ostrum R.F (1996). Journal of orthopaedic trauma..

[15] Jean L (2002). Journal of orthopaedic trauma..

[16] Ahn J (2016). The Journal of foot and ankle surgery..

[17] Bruna B (2013). Revista brasileira de ortopedia..

[18] Jowett A.J.L (2010). Foot & ankle international..

